# Correlation Between Clinical Indicators and Liver Pathology in Children with Chronic Hepatitis B

**DOI:** 10.3390/biomedicines12122903

**Published:** 2024-12-20

**Authors:** Chenyang Huang, Ying Lu, Ziwei Wang, Qiyu Jiang, Yi Dong, Lili Cao, Jianguo Yan, Zhiqiang Xu, Fuchuan Wang, Yinjie Gao, Junliang Fu, Min Zhang, Fu-Sheng Wang

**Affiliations:** 1 Medical School of Chinese PLA, Beijing 100853, China; huangchenyang1990@outlook.com; 2 Department of Infectious Diseases, Fifth Medical Center of Chinese PLA General Hospital, National Clinical Research Center for Infectious Diseases, Beijing 100039, China; wangzwup@163.com (Z.W.); jiangqy1991@sina.com (Q.J.); dong1970yi@sina.com (Y.D.); yanandlili@163.com (L.C.); yan1981jianguo@163.com (J.Y.); fjunliang@163.com (J.F.); 3 Senior Department of Hepatology, Fifth Medical Center of Chinese PLA General Hospital, Beijing 100010, China; luying130406@sina.com (Y.L.); bjxzhq@sina.com (Z.X.); wfc_20002000@163.com (F.W.); gaoyj302@163.com (Y.G.)

**Keywords:** CHB, children, AST, ALT, inflammation, fibrosis

## Abstract

**Background:** Chronic hepatitis B (CHB) in children presents a significant global health challenge, with liver inflammation and fibrosis being critical concerns for disease progression and long-term outcomes. **Methods:** This retrospective study analyzed 1629 pediatric CHB patients from the Fifth Medical Center of Chinese PLA General Hospital, spanning from January 2000 to December 2021. Liver biopsies were performed to assess the severity of liver inflammation and fibrosis, which were graded using the Scheuer scoring system. Key clinical indicators, including age, alanine aminotransferase (ALT), aspartate aminotransferase (AST), and gamma-glutamyl transferase (GGT), were evaluated for their predictive value in determining disease severity using restricted cubic spline regression models. **Results:** Significant nonlinear associations were found between the clinical indicators and liver pathology. Older age was strongly associated with increased risks of moderate to severe inflammation (OR 2.21, 95% CI: 1.34–3.63, *p* = 0.002) and significant fibrosis (OR 2.22, 95% CI: 1.31–3.77, *p* = 0.003). Elevated ALT levels (≥80 U/L) were correlated with a higher likelihood of moderate to severe inflammation (OR 1.82, 95% CI: 1.05–3.15, *p* = 0.033), while higher GGT levels (≥50 U/L) were significantly associated with advanced fibrosis (OR 2.62, 95% CI: 1.72–3.99, *p* < 0.001). **Conclusions:** Regular monitoring of clinical indicators such as ALT, AST, and GGT levels plays a critical role in identifying pediatric CHB patients at higher risk of moderate to severe inflammation and significant fibrosis. Our findings highlight the value of integrating age and key biochemical markers into non-invasive diagnostic algorithms for the early detection and management of liver pathology in children.

## 1. Introduction

Hepatitis B virus (HBV) infection remains a significant public health concern worldwide, with approximately 1.5 million new infections occurring annually. The Polaris Observatory Collaborators estimate that the global hepatitis B surface antigen (HBsAg) prevalence among children under 5 years old was 0.7% in 2022, affecting approximately 5.6 million children [1]. In children, HBV infection often presents with subtler clinical manifestations compared to adults, frequently lacking overt symptoms. However, significant pathological changes can occur in the liver, potentially progressing to fibrosis, cirrhosis, and hepatocellular carcinoma (HCC). According to the Global Burden of Disease (GBD) statistics, as of 2019, around 46.5 million children and adolescents worldwide were living with HBV-related chronic liver diseases [2]. China, bearing the heaviest burden of hepatitis B globally, reported that nearly half of all new HBV cases are acquired during the perinatal period [3]. Furthermore, the prevalence of HBsAg among women of childbearing age in China remains high, highlighting the ongoing challenge of preventing and treating hepatitis B in children [4,5].

HBV infection follows a natural course that varies depending on the age at which infection occurs. In perinatal or early childhood infections, the immune system is often tolerant to the HBV virus, leading to minimal liver inflammation and a high likelihood of developing chronic infection [6]. As children age, the immune system gradually mounts a response against HBV virus, resulting in varying degrees of liver inflammation and damage. This immune response can lead to significant liver pathology, including fibrosis and cirrhosis, particularly in those who remain untreated [7]. Evaluating the level of inflammatory activity and the stage of fibrosis within liver tissue is a crucial clinical tool. Liver biopsy, the gold standard for diagnosing liver diseases, primarily evaluates the degree of liver inflammation, necrosis, and fibrosis. It is essential for monitoring disease progression, determining prognosis, and deciding on treatment initiation [8]. Hu et al. [9] reported a significant proportion of untreated chronic hepatitis B (CHB) children in Shanghai exhibiting moderate to severe hepatic inflammation, fibrosis, or cirrhosis. Notably, when liver biopsy was performed on such children ≤3 years old with alanine aminotransferase (ALT) levels exceeding 80 U/L, the prevalence of moderate fibrosis and cirrhosis surpassed 80%, underscoring the severity of the condition. Liang et al. conducted a cohort study involving liver biopsy analysis of 80 pediatric patients and found that those with fluctuating ALT levels exhibited a higher incidence of hepatic inflammation, which intensified with age [10]. The pathogenesis of HBV infection involves a complex interplay between the virus and the host’s immune system. HBV is a DNA virus that infects hepatocytes, leading to viral replication and the production of viral proteins that can trigger immune responses. The extent and nature of the immune response largely determine the clinical outcomes. In children, the immune tolerance phase can last for years, during which the virus replicates with minimal liver damage. However, as the immune response is activated, liver inflammation can ensue, leading to progressive fibrosis and, in severe cases, cirrhosis and HCC [11].

While previous studies [12,13,14,15,16,17] have provided insights into the pathological characteristics of children with CHB, they are limited by factors such as study design, diagnostic criteria, assessment methodologies, and variations in patient demographics (including geography, age, and disease duration), resulting in considerable heterogeneity. Consequently, these studies struggle to comprehensively capture the true pathological status and disease characteristics of in children with CHB. Against this backdrop, our study aims to analyze the clinical characteristics of liver inflammation and fibrosis in CHB children, explore the influencing factors of liver pathological changes, and assist in the clinical diagnosis and treatment of CHB children.

By refining and expanding upon the current understanding of CHB in children, this study seeks to bridge existing gaps in knowledge and contribute to more effective and targeted clinical management strategies for this vulnerable population. Through comprehensive analysis and advanced diagnostic approaches, this research aspires to enhance the early identification and treatment of liver pathology in children with CHB, ultimately improving their long-term health outcomes.

## 2. Methods and Methods

### 2.1. Patients

A retrospective study was conducted to include pediatric patients admitted to the Fifth Medical Center of Chinese PLA General Hospital from January 2000 to December 2021. The diagnosis of chronic hepatitis B in children was made according to the Expert Consensus on the Prevention and Treatment of Chronic Hepatitis B in Children [18]. The inclusion criteria were as follows: (1) age less than 18 years; (2) positive for hepatitis B surface antigen (HBsAg); (3) complete liver biopsy performed; (4) no antiviral therapy within six months prior to liver biopsy and no history of taking hepatoprotective medications within three months prior to biopsy; and (5) parental written informed consent. Exclusion criteria were (1) co-infection with other hepatitis viruses, alcoholic liver disease, autoimmune liver disease, drug-induced liver injury, liver cancer, and decompensated cirrhosis; (2) HIV infection, malignancy, or other severe systemic diseases; (3) congenital diseases; (4) antiviral or other immunomodulatory drug treatments within six months prior to inclusion; and (5) missing clinical data or incomplete records.

### 2.2. Ethical Clearance and Informed Consent for Participation

This study adhered to the ethical principles outlined in the Declaration of Helsinki [19] and its subsequent amendments, ensuring compliance with internationally recognized ethical norms. Prior to the study, we submitted a detailed research protocol to the Medical Ethics Committee of the Fifth Medical Center of Chinese PLA General Hospital and obtained formal ethical approval (Approval Number: 2020053D). Informed consent was obtained from the parents or legal guardians of all patients included in the study. The consent process was designed to ensure that all participants and their families were fully informed about the study’s purpose, procedures, potential risks, and benefits.

### 2.3. Demographic and Clinical Characteristics

Clinical data were collected by reviewing the electronic medical record system, including demographic information, blood routine tests, biochemical tests, HBV-DNA levels, hepatitis B serological markers, and liver biopsy results. Demographic information included age, gender, and mode of hepatitis B transmission. Biochemical tests included alanine aminotransferase (ALT), aspartate aminotransferase (AST), albumin, alkaline phosphatase (ALP), gamma-glutamyltransferase (GGT), cholinesterase, direct bilirubin, indirect bilirubin, creatinine, uric acid, and alpha-fetoprotein (AFP). All serological reports were tested and issued by a certified laboratory using the same standardized detection methods. Routine blood indicators were tested by an XN2000 automatic blood cell analyzer (Sysmex Corporation, Kobe, Japan). Hepatitis B virus markers were detected using quantitative reagent kits (chemiluminescence method, Roche Diagnostics GmbH, Shanghai, China). HBV DNA viral load was tested using hepatitis B virus reagent kits (high-sensitivity PCR fluorescence probe method, Shengxiang Biotechnology Co., Ltd., Changsha, China).

### 2.4. Histological Examination

Pathological data of the liver were extracted from the pathology system of the Fifth Medical Center of the Chinese PLA General Hospital. All patients undergoing liver tissue pathological examination signed informed consent forms prior to surgery. The biopsy procedure was performed by an experienced sonographer under ultrasound guidance through the intercostal space. An 18 × 16 cm semi-automatic biopsy needle (Bard Medical, Delran, NJ, USA) was used to ensure safety and accuracy. Liver tissue samples (2–3 strips, each approximately 2 cm in length) were fixed with 10% neutral formalin, routinely embedded in paraffin, and subjected to routine HE and hepatitis B-related immunohistochemical examinations. Observations were made under a light microscope.

The biopsy samples were sent to the pathology department for diagnosis, reviewed by pathology experts, and confirmed by at least three pathologists. The entire slide-reading process was conducted in a double-blind manner. According to the Scheuer scoring system [20], inflammation (grade) was categorized into G0, G1, G2, G3, and G4, with G ≥ 2 indicating significant inflammation. Fibrosis stage was classified into S0, S1, S2, S3, and S4, with S ≥ 2 defined as significant fibrosis and S4 representing early cirrhosis. To ensure consistency and accuracy, all biopsy samples were processed and evaluated using standardized protocols, and quality control measures were implemented throughout the histological examination process [21].

### 2.5. Statistical Analysis

Continuous variables were described using medians and interquartile ranges [(P25–P75)], while categorical variables were presented as frequencies (n%). Data preprocessing included handling missing data using multiple imputation methods and identifying outliers using the IQR method. To compare differences between groups for continuous variables, the Mann–Whitney U test was employed due to its robustness in handling non-parametric data or data with unequal variances. For categorical variables, chi-square (χ^2^) tests or Fisher’s exact probability method were utilized.

To control for potential confounders and ensure an accurate comparison between significant inflammation (G ≥ 2) and non-significant inflammation (G < 2) groups as well as significant fibrosis (S ≥ 2) and non-significant fibrosis (S < 2) groups, we utilized propensity score matching (PSM) [22]. Propensity scores were calculated using logistic regression models including variables such as age, sex, genotype, HBeAg status, platelet count (PLT), hemoglobin (HB), red blood cell count (RBC), white blood cell count (WBC), alpha-fetoprotein (AFP), total bilirubin (TBiL), direct bilirubin (DBiL), aspartate aminotransferase (AST), alanine aminotransferase (ALT), alkaline phosphatase (ALP), albumin (ALB), and gamma-glutamyl transferase (GGT). In this study, “age” specifically refers to “age at biopsy”, indicating the age of the patient at the time of the liver biopsy procedure. Nearest neighbor matching with a 1:1 ratio was performed to achieve balance between groups, which was confirmed by comparing standardized mean differences (SMDs). Potential influencing factors of significant liver inflammation (G ≥ 2) and significant fibrosis (S ≥ 2) were screened by single-factor logistic regression, and the odds ratios (OR) and 95% confidence intervals (CIs) were calculated by multi-factor logistic regression, adjusting for potential confounders such as age, gender, and ALT levels. Sensitivity analyses were performed to ensure the robustness of the findings.

Post hoc power analysis was conducted to estimate the study’s power to detect the observed effect sizes [23]. All statistical analyses were conducted using R software (R version 4.2.3; RStudio, Boston, MA, USA), a versatile and powerful tool for statistical computing and graphics.

## 3. Results

### 3.1. Propensity Score Matching

Before matching, the study included approximately 2327 pediatric CHB patients. To control for potential confounders and ensure a more accurate comparison between significant inflammation (G ≥ 2) and non-significant inflammation (G < 2) groups as well as significant fibrosis (S ≥ 2) and non-significant fibrosis (S < 2) groups, we utilized propensity score matching (PSM). The propensity scores were calculated using a logistic regression model that included the following variables: age, sex, genotype, HBeAg status, platelet count (PLT), hemoglobin (HB), red blood cell count (RBC), white blood cell count (WBC), alpha-fetoprotein (AFP), total bilirubin (TBiL), direct bilirubin (DBiL), aspartate aminotransferase (AST), alanine aminotransferase (ALT), alkaline phosphatase (ALP), albumin (ALB), and gamma-glutamyl transferase (GGT).

Using nearest neighbor matching with a 1:1 ratio, we achieved balance between the groups for inflammation and fibrosis. After propensity score matching, the sample sizes were reduced to 1629 for both inflammation and fibrosis, ensuring a more balanced comparison across the covariates. The summary statistics of the matched data are presented in Table 1.

### 3.2. Basic Demographic Information and Characteristics

The study included 1629 pediatric CHB patients with a median age of 7.0 years (interquartile range (IQR), 3.0–12.0 years). The cohort comprised 66.7% males and 33.3% females. Genotype distribution revealed that 37.1% had genotype B, while 62.9% had genotype C. A significant majority, 96.1%, were HBeAg-positive, with only 3.9% being HBeAg-negative. The median HBV-DNA level was 6.88 log IU/mL (IQR, 5.96–7.55 log IU/mL). Other laboratory findings included a median platelet (PLT) count of 247 × 10^9^/L (IQR, 200–296 × 10^9^/L), hemoglobin (HB) level of 129 g/L (IQR, 123–138 g/L), red blood cell (RBC) count of 4.60 × 10^12^/L (IQR, 4.35–4.87 × 10^12^/L), and white blood cell (WBC) count of 6.70 × 10^9^/L (IQR, 5.37–8.50 × 10^9^/L) (Table 2).

### 3.3. Grading of Liver Inflammation and Staging of Fibrosis

**Figure 1:**
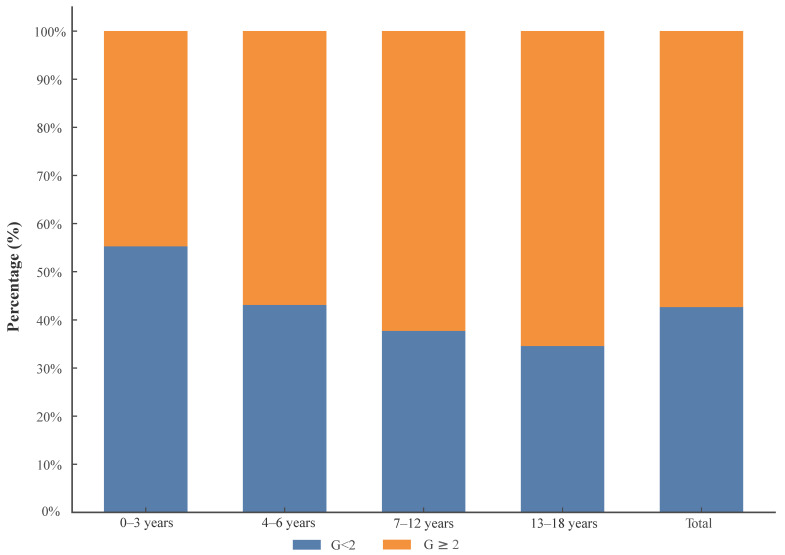
Grading of Liver Inflammation by Age Groups.

**Figure 2:**
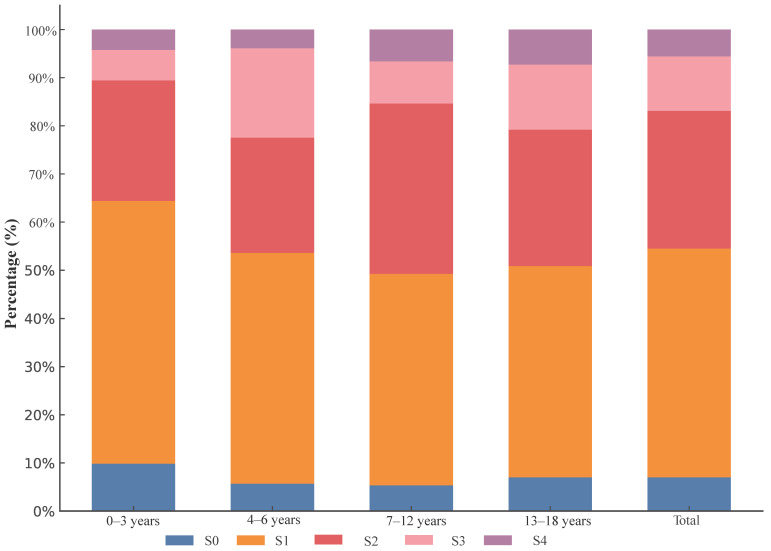
Staging of Liver Fibrosis by Age Groups.

In terms of liver inflammation grading, 55.3% of patients aged 0–3 years exhibited mild inflammation (G < 2), while 44.7% had moderate to severe inflammation (G ≥ 2) (Table 3). For patients aged 4–6 years, mild inflammation was observed in 43.1%, and 56.9% had moderate to severe inflammation. Among those aged 7–12 years, 37.7% had mild inflammation, and 62.3% had moderate to severe inflammation. In the 13–18-years-old age group, 34.6% exhibited mild inflammation, whereas 65.4% showed moderate to severe inflammation. Overall, across all age groups, 42.6% of patients had mild inflammation, and 57.4% had moderate to severe inflammation (Figure 1). For patients aged 0–3 years, fibrosis staging distribution was 9.8% in S0, 54.6% in S1, 25.1% in S2, 6.3% in S3, and 4.2% in S4. In the 4–6-years-old group, 5.7% were in S0, 47.9% in S1, 24.0% in S2, 18.6% in S3, and 3.9% in S4. Among those aged 7–12 years, 5.3% were in S0, 43.9% in S1, 35.4% in S2, 8.7% in S3, and 6.6% in S4. For patients aged 13–18 years, the distribution was 7.0% in S0, 43.9% in S1, 28.3% in S2, 13.5% in S3, and 7.3% in S4. Overall, 7.0% were in S0, 47.5% in S1, 28.6% in S2, 11.3% in S3, and 5.6% in S4 (Figure 2).

### 3.4. Power Analysis Results

To assess the robustness of our study findings, we conducted post hoc power analyses for both the grading of liver inflammation and the staging of fibrosis. Assuming a small effect size (Cohen’s d = 0.3) and with a significance level (alpha) of 0.05, the analysis revealed that the study had a statistical power of 0.9998 for both the grading of liver inflammation and the staging of fibrosis. This high power indicates a strong likelihood that the study could detect a significant effect if one exists, minimizing the risk of Type II error.

### 3.5. Correlation Analysis of Inflammation and Fibrosis with Clinical Indicators

Univariate and multivariate logistic regression analyses were conducted to identify factors associated with moderate to severe inflammation (G ≥ 2) and significant fibrosis (S ≥ 2). The results are presented in Table 4 and Table 5.

In the univariate analysis for inflammation, age, genotype, HBV-DNA levels, PLT, RBC, TBiL, DBiL, AST, ALT, and GGT were significantly associated with moderate to severe inflammation. Patients aged 4–6 years had 1.82 times the odds (95% CI: 1.20–2.76, *p* = 0.005) of having moderate to severe inflammation compared to those aged 0–3 years. Similarly, patients aged 7–12 years and 13–18 years had higher odds of moderate to severe inflammation, with ORs of 2.21 (95% CI: 1.46–3.35, *p* < 0.001) and 1.67 (95% CI: 1.07–2.60, *p* = 0.025), respectively.

In the multivariate analysis, age, genotype, HBV-DNA levels, PLT, RBC, TBiL, DBiL, AST, ALT, and GGT remained significant predictors. Notably, patients aged 4–6 years had 2.21 times the odds (95% CI: 1.34–3.63, *p* = 0.002) and those aged 7–12 years 1.82 times the odds (95% CI: 1.05–3.15, *p* = 0.033) of moderate to severe inflammation compared to those aged 0–3 years. Genotype C and higher levels of TBiL, DBiL, AST, ALT, and GGT were also independently associated with moderate to severe inflammation.

For significant fibrosis, the univariate analysis identified age, genotype, HBV-DNA levels, PLT, RBC, TBiL, DBiL, AST, ALT, and GGT as significant factors. Patients aged 4–6 years had 1.65 times the odds (95% CI: 1.08–2.52, *p* = 0.021) of significant fibrosis compared to those aged 0–3 years. Similarly, patients aged 7–12 years had 2.62 times the odds (95% CI: 1.72–3.99, *p* < 0.001).

In the multivariate analysis, age, genotype, PLT, RBC, WBC, and GGT remained significant. Patients aged 4–6 years had 1.82 times the odds (95% CI: 1.12–2.96, *p* = 0.015) and those aged 7–12 years 2.22 times the odds (95% CI: 1.31–3.77, *p* = 0.003) of significant fibrosis compared to those aged 0–3 years. Genotype C, lower RBC levels, and higher GGT levels were also independently associated with significant fibrosis.

### 3.6. RCS Plots for Predictors of Inflammation and Fibrosis

**Figure 3:**
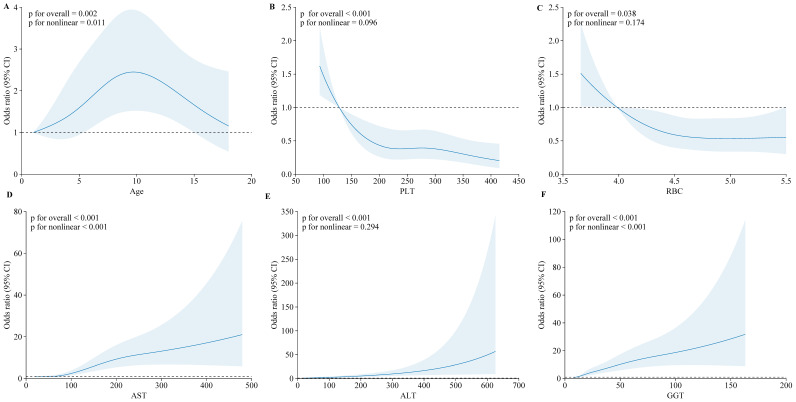
Restricted cubic spline (RCS) analysis of moderate to severe liver inflammation in CHB children. The odds ratio (95% CI) is shown for the following clinical indicators: (**A**) Age; (**B**) Platelet count (PLT); (**C**) Red blood cell count (RBC); (**D**) Aspartate aminotransferase (AST); (**E**) Alanine aminotransferase (ALT); (**F**) Gamma-glutamyl transferase (GGT).

The restricted cubic spline (RCS) regression models highlighted significant nonlinear relationships between key clinical indicators and the odds of moderate to severe liver inflammation (G ≥ 2) and significant fibrosis (S ≥ 2) in children with CHB. For liver inflammation, age and AST were notable predictors. The odds ratio (OR) for moderate to severe inflammation increased significantly with age, particularly after 10 years (*p* for overall = 0.002; *p* for nonlinear = 0.011), indicating that older children are at a higher risk. Similarly, AST levels showed a strong nonlinear relationship with inflammation, with the risk escalating markedly at levels above 100 U/L (*p* for overall < 0.001; *p* for nonlinear < 0.001), underscoring AST as a critical marker for liver inflammation (Figure 3).

**Figure 4:**
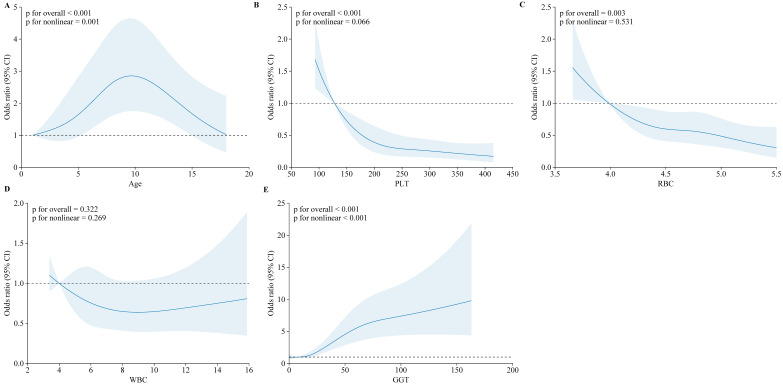
Restricted cubic spline (RCS) analysis of moderate to severe liver fibrosis in CHB children. The odds ratio (95% CI) is shown for the following clinical indicators:(**A**) Age; (**B**) Platelet count (PLT); (**C**) Red blood cell count (RBC); (**D**) White Blood Cell (WBC); (**E**) Gamma-glutamyl transferase (GGT).

For significant fibrosis, age and GGT were the most influential predictors. The odds of significant fibrosis increased with age, with a pronounced rise after 7 years (*p* for overall < 0.001; *p* for nonlinear = 0.001), suggesting that older children are more susceptible to advanced fibrosis. GGT levels demonstrated a robust nonlinear association with fibrosis, with higher levels correlating with increased odds, especially beyond 50 U/L (*p* for overall < 0.001; *p* for nonlinear < 0.001). These findings emphasize the importance of continuous monitoring of these clinical indicators to effectively manage and mitigate the progression of liver disease in pediatric CHB patients (Figure 4).

## 4. Discussion

This study, encompassing a large cohort of 1629 pediatric patients with CHB, represents one of the most comprehensive analyses to date, examining a broad spectrum of clinical and biochemical indicators. The extensive dataset, which includes ALT, AST, GGT, and other liver function tests, provides a robust foundation for understanding the progression of liver disease in this vulnerable population. The uniqueness of our study lies in its scale and the detailed evaluation of multiple clinical indicators, offering novel insights into the early detection and management of liver inflammation and fibrosis in children with CHB.

Age was identified as a significant predictor of both liver inflammation and fibrosis in this study, with older children demonstrating a markedly higher risk. The observed nonlinear relationship between age and disease severity, particularly highlighted by the RCS models, suggests that certain age thresholds are critical in determining the likelihood of severe liver outcomes. This finding aligns with previous research indicating that older age in CHB patients is associated with a more aggressive disease course, possibly due to the cumulative effects of prolonged HBV exposure and an evolving immune response [24]. The maturation of the immune system, particularly the adaptive immune response, could result in an intensified reaction to the virus in older children, leading to more severe inflammation and fibrosis. These age-related dynamics highlight the importance of early and sustained monitoring in pediatric CHB patients, with a focus on tailored intervention strategies as children approach these critical periods.

Alanine aminotransferase (ALT) and aspartate aminotransferase (AST) are central to the assessment of liver health, particularly in the context of CHB. In our study, ALT levels emerged as a pivotal marker, correlating strongly with both the degree of liver inflammation and fibrosis. Elevated ALT levels, particularly those exceeding 80 U/L, were associated with significant hepatic injury, underscoring ALT’s role as a frontline biomarker in monitoring disease progression [9]. However, it is crucial to acknowledge that ALT, while specific to hepatocellular damage, may not fully capture the complexity of liver pathology, as it does not differentiate between inflammation and fibrosis, nor does it provide insight into the underlying mechanisms driving these processes [25]. Therefore, relying solely on ALT levels may overlook the underlying severity of liver pathology. Given this, performing a liver biopsy in children with abnormal ALT levels becomes crucial [26].

AST, although less specific to the liver than ALT, provides complementary information. Our data revealed that elevated AST levels were significantly associated with advanced fibrosis and the progression to cirrhosis. This is consistent with the understanding that AST, being partially localized in mitochondria, is more indicative of severe and chronic liver damage, where mitochondrial dysfunction plays a key role [27]. The combination of elevated ALT and AST [28], particularly when the AST/ALT ratio exceeds 1, has been suggested as a marker for more advanced fibrosis, reflecting the extent of hepatocellular injury and the fibrotic response. This ratio’s significance in predicting fibrosis underscores the need for comprehensive liver function assessment in pediatric CHB patients, particularly in those with prolonged disease duration or higher viral loads.

The elevation of ALT and AST in CHB is driven by the hepatocellular damage induced by the hepatitis B virus (HBV) and the host immune response. HBV directly damages liver cells through viral replication and integration into the host genome, leading to cytopathic effects. Additionally, the immune-mediated destruction of infected hepatocytes, particularly by cytotoxic T lymphocytes, exacerbates liver injury, further elevating ALT and AST levels [29]. Persistent inflammation and the activation of hepatic stellate cells contribute to fibrosis, a process in which AST, due to its mitochondrial association, serves as a marker of ongoing cellular stress and damage [30]. Moreover, AST’s association with more severe liver pathology, including cirrhosis, highlights the enzyme’s potential role in not only diagnosing but also in monitoring disease progression and treatment efficacy.

Gamma-glutamyl transferase (GGT) is another critical enzyme that reflects biliary damage and fibrosis. Elevated GGT levels in our cohort were strongly associated with more severe fibrosis and the development of cirrhosis, highlighting its utility as a non-invasive marker for advanced liver disease. The pathophysiology underlying GGT elevation in CHB includes bile duct damage and cholestasis, both of which are exacerbated by the chronic inflammatory milieu created by HBV infection [21]. The inflammatory response induced by HBV not only targets hepatocytes but also affects the biliary system, leading to secondary damage such as bile duct proliferation and fibrosis. These processes result in elevated GGT levels, making it a reliable indicator of more severe liver disease in children with CHB. The combination of GGT with ALT and AST provides a more comprehensive picture of liver health, particularly in identifying patients at risk of progressing to cirrhosis. Given the association between GGT and advanced liver pathology, its role in routine clinical practice should be emphasized. Regular monitoring of GGT, alongside ALT and AST, can help identify children at higher risk for severe outcomes, allowing for timely referral for specialized care and consideration of antiviral therapy. The inclusion of GGT in non-invasive scoring systems could enhance the predictive accuracy for fibrosis and cirrhosis, especially in resource-limited settings where liver biopsy is not feasible [13]. Additionally, integrating GGT into existing predictive models could improve early detection of biliary complications, guiding more targeted and effective therapeutic interventions.

The findings from this study have significant clinical implications for the management of pediatric CHB. The identification of key clinical indicators such as ALT, AST, and GGT as predictors of liver disease progression underscores the need for their regular monitoring. These markers can help stratify patients based on their risk of developing significant fibrosis or cirrhosis, thereby guiding clinical decisions regarding the timing and intensity of therapeutic interventions. The strong correlation between these clinical indicators and liver disease severity suggests that they could also serve as endpoints in clinical trials, particularly those evaluating the efficacy of antiviral treatments or novel therapeutic agents aimed at reducing inflammation and fibrosis in pediatric populations.

Despite the strengths of this study, including its large sample size and comprehensive biomarker analysis, several limitations must be acknowledged. The retrospective nature of the study may introduce selection bias, and the lack of longitudinal follow-up limits our ability to fully understand the temporal progression of liver disease in these patients. Additionally, while ALT, AST, and GGT are valuable clinical indicators, their levels can be influenced by various factors, including concurrent infections or medications, which may confound their interpretation. Future studies should aim to validate these findings in prospective cohorts and explore the integration of additional non-invasive markers, such as elastography or serum fibrosis panels, to enhance the accuracy of liver disease staging in pediatric CHB. These advanced diagnostic tools could complement traditional clinical indicators, providing a more nuanced understanding of disease progression and facilitating earlier intervention. Our study revealed that children with CHB genotype C exhibited more severe liver fibrosis and inflammation compared to those with other genotypes in our study, which is consistent with observations in adults [31].

In addition, the relationship between PLT and liver fibrosis demonstrates a certain degree of nonlinearity (*p* for overall < 0.001, *p* for nonlinear = 0.666). However, when PLT levels are either high or low, the risk of fibrosis in children with chronic hepatitis B does not significantly increase or decrease. In contrast, PLT is one of the key factors in assessing liver fibrosis in adults [32]. Similarly, RBC shows a nonlinear trend (*p* for overall = 0.003, *p* for nonlinear = 0.531), but its changes are not pronounced in these pediatric patients, indicating that RBC responses vary across different stages or among different patients with chronic hepatitis B-associated fibrosis.

Furthermore, the role of emerging clinical indicators and novel technologies, such as liver stiffness measurement and fibrosis clinical indicators, should be explored to complement traditional liver function tests. These tools may offer more precise assessments of liver fibrosis and inflammation, potentially leading to better-tailored treatment strategies for pediatric CHB patients. Additionally, further research is needed to investigate the potential of combining these non-invasive methods with routine laboratory tests, aiming to develop comprehensive, cost-effective diagnostic algorithms that could be easily implemented in clinical practice. This approach could significantly improve the management and outcomes of children with CHB, particularly in settings with limited access to advanced diagnostic facilities.

Furthermore, another limitation is as follows: As all cases included in this study underwent liver biopsy based on clinical need, such as the need for antiviral therapy, there may be a potential selection bias. This could limit the generalizability of our findings to population-based cohorts. Liver biopsy is generally performed for specific clinical needs rather than routinely applied to all suspected cases, which may affect the representativeness of our sample. Future studies could address this limitation by incorporating non-invasive diagnostic techniques or population-based screening to provide a broader understanding of pediatric CHB. In addition, due to the inability to accurately trace the infection history of patients, we were unable to directly evaluate the duration of HBV infection.

## 5. Conclusions

In conclusion, this study provides a comprehensive evaluation of ALT, AST, and GGT as clinical indicators of liver disease in children with CHB. The large dataset and detailed analysis underscore the importance of these enzymes in the early detection of liver inflammation and fibrosis, offering critical insights into the management of pediatric CHB. However, further research is needed to refine these clinical indicators’ utility and explore additional diagnostic tools to improve the early identification and treatment of advanced liver disease in this population.

## Figures and Tables

**Table 1:** Parameter of Propensity Score Matching for Significant Liver Inflammation and Fibrosis in Children with CHB.

Variable	S0–1 Mean (SD) Pre-Match	S2–4 Mean (SD) Pre-Match	G0–1 Mean (SD) Post-Match	G2–4 Mean (SD) Post-Match	Fibrosis SMD Pre-Match	Fibrosis SMD Post-Match	G0–1 Mean (SD) Pre-Match	G2–4 Mean (SD) Pre-Match	G0–1 Mean (SD) Post-Match	G2–4 Mean (SD) Post-Match	Inflammation SMD Pre-Match	Inflammation SMD Post-Match
Age (years)	6.5 (3.1)	7.2 (3.0)	6.8 (3.1)	6.9 (3.0)	5:31:12	1:12:00	6.5 (3.1)	7.2 (3.0)	6.8 (3.1)	6.9 (3.0)	5:31:12	1:12:00
Sex (% male)	66.3 (0.47)	68.2 (0.47)	67.1 (0.47)	67.8 (0.47)	0:57:36	0:28:48	66.3 (0.47)	68.2 (0.47)	67.1 (0.47)	67.8 (0.47)	0:57:36	0:28:48
Genotype B (%)	34.2 (0.48)	35.6 (0.48)	34.9 (0.48)	35.2 (0.48)	0:43:12	0:14:24	34.2 (0.48)	35.6 (0.48)	34.9 (0.48)	35.2 (0.48)	0:43:12	0:14:24
HBeAg (% positive)	55.0 (0.50)	53.0 (0.50)	54.0 (0.50)	54.5 (0.50)	0:57:36	0:14:24	55.0 (0.50)	53.0 (0.50)	54.0 (0.50)	54.5 (0.50)	0:57:36	0:14:24
HBV-DNA (lgIU/mL)	4.7 (1.2)	5.0 (1.2)	4.8 (1.2)	4.9 (1.2)	6:00:00	1:55:12	4.7 (1.2)	5.0 (1.2)	4.8 (1.2)	4.9 (1.2)	6:00:00	1:55:12
PLT (10^9^/L)	231.2 (54.6)	224.7 (56.3)	229.5 (55.2)	227.4 (55.9)	2:52:48	0:57:36	231.2 (54.6)	224.7 (56.3)	229.5 (55.2)	227.4 (55.9)	2:52:48	0:57:36
HB (g/L)	130.0 (15.0)	128.5 (14.5)	129.8 (14.8)	129.1 (14.6)	2:24:00	0:28:48	130.0 (15.0)	128.5 (14.5)	129.8 (14.8)	129.1 (14.6)	2:24:00	0:28:48
RBC (10^9^/L)	4.5 (0.6)	4.4 (0.5)	4.5 (0.6)	4.5 (0.5)	4:48:00	1:12:00	4.5 (0.6)	4.4 (0.5)	4.5 (0.6)	4.5 (0.5)	4:48:00	1:12:00
WBC (10^12^/L)	5.5 (1.2)	5.4 (1.1)	5.5 (1.2)	5.5 (1.1)	3:36:00	0:43:12	5.5 (1.2)	5.4 (1.1)	5.5 (1.2)	5.5 (1.1)	3:36:00	0:43:12
AFP (ng/mL)	10.0 (5.0)	10.5 (4.8)	10.2 (4.9)	10.3 (4.7)	1:55:12	0:28:48	10.0 (5.0)	10.5 (4.8)	10.2 (4.9)	10.3 (4.7)	1:55:12	0:28:48
TBiL (μmol/L)	20.0 (5.0)	21.5 (5.2)	20.8 (5.1)	21.0 (5.0)	4:19:12	0:57:36	20.0 (5.0)	21.5 (5.2)	20.8 (5.1)	21.0 (5.0)	4:19:12	0:57:36
DBiL (μmol/L)	5.0 (1.5)	5.2 (1.6)	5.1 (1.5)	5.1 (1.6)	2:24:00	0:43:12	5.0 (1.5)	5.2 (1.6)	5.1 (1.5)	5.1 (1.6)	2:24:00	0:43:12
AST (U/L)	45.0 (10.0)	46.0 (9.5)	45.5 (9.8)	45.8 (9.6)	2:24:00	0:28:48	45.0 (10.0)	46.0 (9.5)	45.5 (9.8)	45.8 (9.6)	2:24:00	0:28:48
ALT (U/L)	50.0 (15.0)	51.5 (14.5)	50.8 (14.8)	51.0 (14.6)	3:36:00	0:43:12	50.0 (15.0)	51.5 (14.5)	50.8 (14.8)	51.0 (14.6)	3:36:00	0:43:12
ALP (U/L)	80.0 (20.0)	82.5 (19.5)	81.8 (19.8)	82.0 (19.6)	3:07:12	0:57:36	80.0 (20.0)	82.5 (19.5)	81.8 (19.8)	82.0 (19.6)	3:07:12	0:57:36
ALB (g/L)	40.0 (5.0)	39.5 (4.8)	39.8 (4.9)	39.7 (4.7)	1:55:12	0:28:48	40.0 (5.0)	39.5 (4.8)	39.8 (4.9)	39.7 (4.7)	1:55:12	0:28:48
GGT (U/L)	25.0 (10.0)	26.5 (9.5)	25.8 (9.8)	26.0 (9.6)	4:19:12	0:43:12	25.0 (10.0)	26.5 (9.5)	25.8 (9.8)	26.0 (9.6)	4:19:12	0:43:12

**Table 2:** Basic demographic information and characteristics.

Variables	Range *
Age (years)	7.0 (3.0–12.0)
Sex	
Male	1086 (66.7%)
Female	543 (33.3%)
Genotype	
B	604 (37.1%)
C	1025 (62.9%)
HBeAg	
Positive	1566 (96.1%)
Negative	118 (3.9%)
HBV-DNA (lgIU/mL)	6.88 (5.96–7.55)
PLT (10^9^/L)	247 (200–296)
HB (g/L)	129 (123–138)
RBC (10^12^/L)	4.60 (4.35–4.87)
WBC (×10^9^/L)	6.70 (5.37–8.50)
AFP (ng/mL)	6 (4–11)
TBiL (μmol/L)	6.7 (5.0–9.6)
DBiL (μmol/L)	2.00 (1.30–3.03)
AST (U/L)	72 (49–118)
ALT (U/L)	89.0 (53.4–148.0)
ALP (U/L)	277.1 (231.1–322.2)
ALB (g/L)	42.0 (39.0–44.0)
GGT (U/L)	19 (13–42)

* Frequency (%) or median (25th–75th percentile).

**Table 3:** The grading of liver inflammation and staging of fibrosis in patients of various age groups.

Inflammation Grading	0–3 Years	4–6 Years	7–12 Years	13–18 Years	Total
G0–1	236	144	177	138	695
G2–4	191	190	292	261	934
Fibrosis Staging					
S0	42	19	25	28	114
S1	233	160	206	175	774
S2	107	80	166	113	466
S3	27	62	41	54	184
S4	18	13	31	29	91
Total	427	334	469	399	1629

**Table 4:** Correlation Analysis of Moderate To Severe Inflammation With Clinical Indicators Using Univariate and Multivariate Logistic Regression Models.

Variables	Univariable	*p*	Multivariable	*p*
	OR (95% CI)		OR (95% CI)	
Age (years)				
0–3 years	Reference			
4–6 years	1.82 (1.20–2.76)	0.005	2.21 (1.34–3.63)	0.002
7–12 years	2.21 (1.46–3.35)	<0.001	1.82 (1.05–3.15)	0.033
13–18 years	1.67 (1.07–2.60)	0.025	1.55 (0.84–2.83)	0.158
Sex				
Female	Reference			
Male	1.04 (0.75–1.43)	0.831		
Genotype				
B	Reference			
C	1.87 (1.33–2.63)	<0.001	1.85 (1.25–2.76)	0.002
HBeAg				
Negative	Reference			
Positive	0.47 (0.14–1.56)	0.219		
HBV-DNA (lgIU/mL)	0.86 (0.77–0.96)	0.008	0.88 (0.78–1.01)	0.064
PLT (10^9^/L)	1.00 (0.99–1.00)	<0.001	1.00 (0.99–1.00)	0.007
Hb (g/L)	1.01 (1.00–1.02)	0.232		
RBC (10^9^/L)	0.60 (0.41–0.87)	0.007	0.61 (0.38–0.97)	0.037
WBC (10^12^/L)	0.95 (0.90–1.01)	0.102	1.07 (0.98–1.16)	0.142
AFP (ng/mL)	1.01 (1.00–1.02)	0.052	1.00 (1.00–1.00)	0.57
TBiL (μmol/L)	1.11 (1.06–1.15)	<0.001	1.06 (0.98–1.15)	0.133
DBiL (μmol/L)	1.23 (1.12–1.35)	<0.001	0.94 (0.80–1.11)	0.48
AST (U/L)	1.01 (1.01–1.01)	<0.001	1.01 (1.00–1.01)	0.013
ALT (U/L)	1.01 (1.01–1.01)	<0.001	1.00 (1.00–1.01)	0.023
ALP (U/L)	1.00 (1.00–1.00)	0.203		
ALB (g/L)	1.01 (0.99–1.03)	0.195	1.01 (0.99–1.02)	0.567
GGT (U/L)	1.04 (1.03–1.05)	<0.001	1.02 (1.01–1.03)	<0.001

**Table 5:** Correlation Analysis of Notable Fibrosis With Clinical Indicators Using Univariate and Multivariate Logistic Regression Models.

Variables	Univariable	*p*	Multivariable	*p*
	OR (95% CI)		OR (95% CI)	
Age (years)				
0–3 years	Reference			
4–6 years	1.65 (1.08–2.52)	0.021	1.82 (1.12–2.96)	0.015
7–12 years	2.62 (1.72–3.99)	<0.001	2.22 (1.31–3.77)	0.003
13–18 years	1.46 (0.92–2.29)	0.105	1.03 (0.56–1.88)	0.934
Sex				
Female	Reference			
Male	0.93 (0.68–1.29)	0.674		
Genotype				
B	Reference			
C	2.26 (1.56–3.25)	<0.001	1.80 (1.20–2.69)	0.004
HBeAg				
Negative	Reference			
Positive	0.91 (0.30–2.74)	0.868		
HBV-DNA (lgIU/mL)	0.86 (0.77–0.95)	0.005	0.91 (0.80–1.03)	0.121
PLT (10^9^/L)	0.99 (0.99–1.00)	0.001	1.00 (0.99–1.00)	0.001
Hb (g/L)	1.01 (1.00–1.02)	0.057	1.02 (1.00–1.04)	0.114
RBC (10^9^/L)	0.46 (0.31–0.69)	<0.001	0.37 (0.20–0.68)	0.001
WBC (10^12^/L)	0.94 (0.89–1.00)	0.057	1.10 (1.01–1.20)	0.025
AFP (ng/mL)	1.00 (1.00–1.00)	0.365		
TBiL (μmol/L)	1.10 (1.05–1.14)	<0.001	1.05 (0.97–1.13)	0.235
DBiL (μmol/L)	1.18 (1.09–1.29)	<0.001	0.97 (0.83–1.14)	0.716
AST (U/L)	1.00 (1.00–1.00)	<0.001	1.00 (1.00–1.00)	0.561
ALT (U/L)	1.00 (1.00–1.00)	<0.001	1.00 (1.00–1.00)	0.528
ALP (U/L)	1.00 (1.00–1.00)	0.443		
ALB (g/L)	1.01 (1.00–1.03)	0.142	1.01 (0.99–1.04)	
GGT (U/L)	1.02 (1.02–1.03)	<0.001	1.01 (1.01–1.02)	0.001

## Data Availability

The data supporting the results of this study can be obtained from the corresponding author upon reasonable request.
